# Haplotype Probabilities in Advanced Intercross Populations

**DOI:** 10.1534/g3.111.001818

**Published:** 2012-02-01

**Authors:** Karl W. Broman

**Affiliations:** Department of Biostatistics and Medical Informatics, University of Wisconsin–Madison, Madison, Wisconsin 53706

**Keywords:** advanced intercross lines, heterogeneous stock, diversity outcross, map expansion, Collaborative Cross, Mouse Genetic Resource

## Abstract

Advanced intercross populations, in which multiple inbred strains are mated at random for many generations, have the advantage of greater precision of genetic mapping because of the accumulation of recombination events across the multiple generations. Related designs include heterogeneous stock and the diversity outcross population. In this article, I derive the two-locus haplotype probabilities on the autosome and X chromosome with these designs. These haplotype probabilities provide the key quantities for developing hidden Markov models for the treatment of missing genotype information. I further derive the map expansion in these populations, which is the frequency of recombination breakpoints on a random chromosome.

Advanced intercross populations, in which multiple inbred strains are mated at random for many generations, have the advantage of greater precision of genetic mapping because of the accumulation of recombination events across the multiple generations. The most commonly used form, which begins with two inbred strains, was formally introduced by Darvasi and Soller (1995) and called advanced intercross lines (AIL). A closely related design is that of heterogeneous stock (HS; see Mott *et al.* 2000), in which eight inbred strains are randomly mated for many generations. Svenson *et al.* (2012) developed the diversity outcross population (DO), which was formed with progenitors that were partially inbred individuals drawn from intermediate generations in the development of the Collaborative Cross (so-called pre-CC mice; see Aylor *et al.* 2011).

The mapping of quantitative trait loci in such populations, whether by interval mapping (Lander and Botstein 1989) or Haley-Knott regression (Haley and Knott 1992), generally requires conditional genotype probabilities at putative quantitative trait loci, given the available marker genotype data. Such probabilities are often calculated using a hidden Markov model (HMM; see Broman and Sen 2009, App. D). An HMM for this purpose formally requires the calculation of two-locus diplotype probabilities, although if the populations are formed with a large number of mating pairs, the two haplotypes within an individual are independent, and so it is sufficient to calculate two-locus haplotype probabilities.

Darvasi and Soller (1995) derived the two-locus haplotype probabilities for the autosome in AIL. I am not aware of any work considering the X-chromosome. In this article, I derive the two-locus haplotype probabilities for the autosome and X-chromosome in AIL, HS, and the DO. The calculations for the DO rely on recent results on haplotype probabilities in pre-CC mice (Broman 2012). Throughout, I assume an effectively infinite set of mating pairs at each generation, no sex difference in recombination, and no selection or mutation.

Let us first revisit the two-locus autosomal haplotype probabilities in AIL, as they serve as a simple example of the technique used in these calculations (see also Bulmer 1980, Ch. 3). Let *p_s_* denote the frequency of the *AA* haplotype at generation F_*s*_. Then $p_{1}=\frac{1}{2}$ and we have the recurrence relation

(1) $$p_{s+1}=\left(1-r\right)p_{s}+r\cdot \frac{1}{2}\cdot \frac{1}{2}$$

where *r* is the recombination fraction (in one meiosis) between the two loci. Equation (1) is derived by noting that an *AA* haplotype drawn from generation F_*s*+1_ is either an intact *AA* haplotype at generation F_*s*_, transmitted without recombination, or it is a recombinant haplotype bringing two independent *A* alleles together. Note that the frequency of the *A* allele is $\frac{1}{2}$ at every generation.

The solution of this recurrence relation (see Graham *et al.* 1994) is, for *s* ≥ 2,

(2) $$p_{s}=\frac{1}{4}\left[1+\left(1-2r\right){\left(1-r\right)}^{s-2}\right].$$

The frequency of recombinant haplotypes at generation F_*s*_ is 1 − 2*p_s_*.

For the X-chromosome in AIL, I will first consider a balanced case, begun with equal proportions of F_1_ individuals from reciprocal crosses, *A* × B and *B* × *A*, so that the F_1_ males are equally likely to be hemizygous *A* or *B*. Let *m_s_* and *f_s_* denote the frequency of the AA haplotype in males and females, respectively, at generation F_*s*_. Then $m_{1}=f_{1}=\frac{1}{2}$ and we have

(3) $$\begin{array}{l} m_{s+1}=\left(1-r\right)f_{s}+\frac{r}{4}\\ f_{s+1}=\left(\frac{1}{2}\right)m_{s}+\left(\frac{1-r}{2}\right)f_{s}+\frac{r}{8} \end{array}$$

This recurrence relation is derived in a similar way to that for the autosome, noting that the male haplotype was drawn from his mother, with a chance for recombination, and a random female haplotype is equally likely to have been drawn from her father, without recombination, or from her mother, with the potential for recombination. I again make use of the fact that the frequency of the *A* allele is $\frac{1}{2}$ in both males and females at every generation. The solution to this relation is, for *s* ≥ 2,

(4) $$\begin{array}{l} m_{s}=\frac{1}{8}\left[2+\left(1-2r\right)\left(w^{s-2}+y^{s-2}\right)+\left(\frac{3-5r+2r^{2}}{z}\right)\left(w^{s-2}-y^{s-2}\right)\right]\\ f_{s}=\frac{1}{8}\left[2+\left(1-2r\right)\left(w^{s-2}+y^{s-2}\right)+\left(\frac{3-6r+r^{2}}{z}\right)\left(w^{s-2}-y^{s-2}\right)\right] \end{array}$$

where $z=\sqrt{\left(1-r\right)\left(9-r\right)}$, *w* = (1 − *r* + *z*)/4, and *y* = (1 − *r* − *z*)/4. Note that the frequencies of recombinant haplotypes in males and females are 1 − 2*m_s_* and 1 − 2*f_s_*, respectively, and that the overall frequency is 1 − (2*m_s_* +4*f_s_*)/3.

Now I turn to the unbalanced case for the X-chromosome, in which all F_1_ individuals are derived from the cross female *A* × male *B*, so that all F_1_ males are hemizygous *A*. This appears to be widely used in practice (*e.g.,* Norgard *et al.* 2008; Kelly *et al.* 2010). The calculations are more difficult, because the allele frequencies are different in males and females and across generations.

I first calculate the single-locus allele frequencies. Let *q_s_* be the frequency of the *A* allele in females at generation F_*s*_. Note that the frequency in males at F_*s*_ is *q_s_*_−1_. The initial values are *q*_0_ = 1 and $q_{1}=\frac{1}{2}$, and we have the recurrence relation $q_{s+1}=\frac{1}{2}q_{s}+\frac{1}{2}q_{s-1}$, which comes from the fact that a random allele drawn from the female at generation F_*s*+1_ is equally likely to be an allele from the female or male at generation F_*s*_, and the allele in the male at F_*s*_ is a random allele from the female at F_*s*−1_. The solution of the recurrence relation is $q_{s}=\frac{2}{3}+\left(\frac{1}{3}\right){\left(-\frac{1}{2}\right)}^{s}$, for *s* ≥ 0.

I now turn to the two-locus haplotype probabilities. Let ${m^{\prime }}_{s}$ and ${f^{\prime }}_{s}$ denote the frequencies of the *AA* haplotype on the X chromosome in males and females at generation F_*s*_ in an unbalanced AIL, and note that ${m^{\prime }}_{1}=1$ and ${f^{\prime }}_{1}=\frac{1}{2}$. The haplotype probabilities satisfy a recurrence relation similar to that in equation (3):

(5) $$\begin{array}{l} {m^{\prime }}_{s+1}=\left(1-r\right){f^{\prime }}_{s}+rq_{s-1}q_{s-2}\\ {f^{\prime }}_{s+1}=\left(\frac{1}{2}\right)m_{s}^{\prime }+\left(\frac{1-r}{2}\right){f^{\prime }}_{s}+\left(\frac{r}{2}\right)q_{s-1}q_{s-2} \end{array}$$

Note the distinction between equations (3) and (5): if a recombinant haplotype is transmitted from the F_*s*_ female, the chance that it brings two *A* alleles together depends on the frequency of the *A* allele in males and females in the *F_s_*_−1_ generation. In the balanced case, these are each $\frac{1}{2}$ ; in the unbalanced case, they are different from each other and vary across generations.

I have been unable to obtain closed-form solutions for ${m^{\prime }}_{s}$ and ${f^{\prime }}_{s}$. However, the values can be quickly calculated numerically, using equation (5). Note that $\lim_{s\to \infty }{f^{\prime }}_{s}=\lim_{s\to \infty }{m^{\prime }}_{s}=\frac{4}{9}$.

Haplotype probabilities in the DO are calculated similarly. The progenitors for the DO were pre-CC mice. I assume a large number of progenitors, that they were drawn from independent lines, and that the order of the crosses that generated the different lines were random, giving complete balance across the eight alleles.

In a potential abuse of notation, I will redefine the *q*, *p*, *m*, and *f* variables used previously. Let *q_k_* denote the frequency of the *AA* haplotype at generation G_2_:F_*k*_ in the pre-CC; this is $\frac{1-r}{2}$ times the haplotype probability in Table 4 of Broman (2012). Let *p_s_* be the probability of the *AA* haplotype at generation *s* of the diversity outcross.

The pre-CC progenitors of the DO were drawn from independent lines at a variety of different generations along the course to inbreeding. Let α*_k_* denote the proportion of the pre-CC progenitors that were at generation G_2_: F_*k*_, and note that a pre-CC progenitor at generation G_2_: F_*k*_ will transmit the *AA* haplotype with frequency *q_k_*_+1_ (that is, the frequency of the *AA* haplotype at generation G_2_: F_*k*_). Thus, the frequency of the *AA* haplotype at the first generation of the DO is $p_{1}=\sum_{k}\alpha_{k}q_{k+1}$.

The recurrence relation for the *p_s_* is like that in equation (1): *p_s_*_+1_ = (1 − *r*)*p_s_* + *r*/64. The solution is

(6) $$p_{s}=\frac{1}{64}+{\left(1-r\right)}^{s-1}\left(p_{1}-\frac{1}{64}\right)$$

Note that the recombinant haplotypes are all equally likely, due to the random order of the initial crosses, and so each has probability (1 − 8*p_s_*)/56.

HS corresponds to the DO with α_1_ = 1 (that is, *k* ≡ 1), in which case *p*_1_ = *q*_2_ = 7 − 24*r* + 24*r*^2^ − 8*r*^3^.

I now turn to the X-chromosome. Let *m_s_* and *f_s_* denote the frequency of the *AA* haplotype on the X chromosome in males and females in the DO at generation *s*. Assuming random orders of crosses to generate the pre-CC progenitors,

(7) $$f_{1}=\sum_{k}\alpha_{k}\left(\frac{1}{8}\right)\left[\left(2-r\right)h_{k+1}^{AA}+\left(1-r\right)h_{k+1}^{CC}\right]$$

where $h_{k+1}^{AA}$ and $h_{k+1}^{CC}$ are the frequencies of the *AA* and *CC* haplotypes, respectively, on the X-chromosome in females at generation G_1_: F_*k*+1_ in the construction of four-way RIL by sibling mating (see Broman 2012, Table 4). *m*_1_ is calculated in the same way. The recurrence relations are much like equation (3):

(8) $$\begin{array}{l} m_{s+1}=\left(1-r\right)f_{s}+\frac{r}{64}\\ f_{s+1}=\left(\frac{1}{2}\right)m_{s}+\left(\frac{1-r}{2}\right)f_{s}+\frac{r}{128} \end{array}$$

The solutions are the following:

(9) $$\begin{array}{l} m_{s}=\frac{1}{128}\left\{2+\left[\frac{\left(64m_{1}-256f_{1}+3\right)\left(1-r\right)}{z}\right]\left(y^{s-1}-w^{s-1}\right)-\left(1-64m_{1}\right)\left(w^{s-1}+y^{s-1}\right)\right\}\\ f_{s}=\frac{1}{128}\left\{2+\left[\frac{-64f_{1}\left(1-r\right)-128m_{1}+3-r}{z}\right]\left(y^{s-1}-w^{s-1}\right)-\left(1-64f_{1}\right)\left(w^{s-1}+y^{s-1}\right)\right\} \end{array}$$

where *w*, *y*, and *z* are as in equation (4).

Again, HS corresponds to DO with α_1_ = 1, in which case *f*_1_ = (4 − 5*r* + *r*^2^)/32 and, *m*_1_ = (2 − 3*r* + *r*^2^)/16.

In Figure 1, the probabilities of recombinant two-locus haplotypes are displayed for the different populations. For the DO, I used the distribution of *k* as in Figure 1 of Svenson *et al.* (2012) and *s* = 5. For HS and AIL, I used *s* = 10 and 12, respectively, to match the total number of generations with recombination—the average *k* in Svenson *et al.* (2012) was six. Recombinant haplotypes are more frequent on the autosome, and are more frequent in HS than in the DO; inbreeding in the pre-CC progenitors of the DO is accompanied by a loss of recombinants.

**Figure 1:**
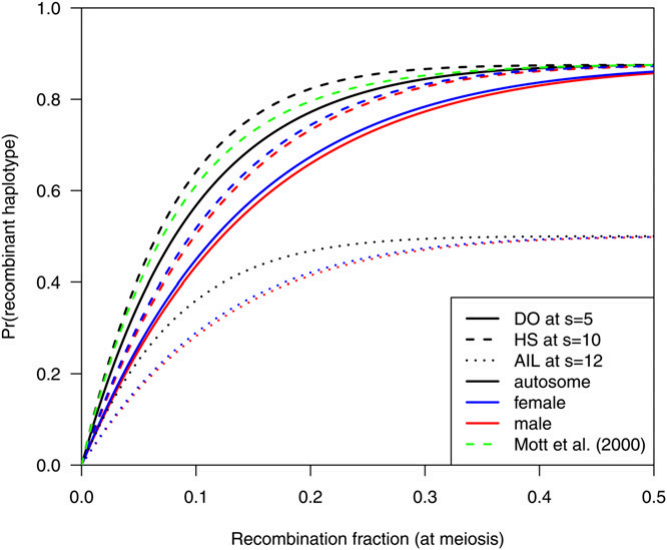
Frequency of a two-locus haplotype being recombinant, as a function of the recombination fraction at meiosis, for the diversity outcross population at *s* = 5 (solid curves), heterogeneous stock at *s* = 10 (dashed curves), and balanced AIL at *s* = 12 (dotted curves), for the autosome (black), male X (blue), and female X (red). The green dashed curve is the recombinant frequency for HS at *s* = 10 assumed in Mott *et al*. (2000).

It is particularly interesting to consider the map expansion in these populations, which is the frequency of recombination breakpoints on a random chromosome. Let *R* denote the probability of a recombinant haplotype; then the map expansion is ${\frac{dR}{dr}|}_{r=0}$ (see Teuscher and Broman 2007). The map expansion on an autosome in AIL is *s*/2. For the DO, on an autosome, the map expansion satisfies $M_{s}=\frac{7}{8}\left(s-1\right)+M_{1}$, where *M*_1_ is the weighted average (with weights α_*k*_) of the map expansion in the pre-CC at generation G_2_: F_*k*+1_ (see Broman 2012, Table 4). For the particular progenitors detailed in Svenson *et al.* (2012, Figure 1), this is approximately (7*s* +37)/8. For HS, we have *M*_1_ = 3 and $M_{s}=\frac{7s+17}{8}$.

For the X-chromosome in balanced AIL, HS and DO, the map expansion is $\frac{2}{3}$ that of the autosome. For the case of the X-chromosome in unbalanced AIL, in which all F_1_ males are hemizygous *A*, I cannot derive a closed-form solution, but taking the derivatives of the recurrence relations in equation (5), I can derive a simple recurrence relation for the map expansion. (Note that the overall map expansion on the X-chromosome can be obtained as the average of the sex-specific map expansions, with $\frac{2}{3}$ weight given to the female, since two-thirds of the X-chromosomes are in females.) Let ${M^{\prime }}_{s}$ denote the map expansion at F_*s*_, and again let *q_s_* be the frequency of the *A* allele in females at F_*s*_. Then we have

(10) $${M^{\prime }}_{s+1}={M^{\prime }}_{s}+\frac{4}{3}\left(q_{s}-q_{s-1}q_{s-2}\right)$$

with the initial conditions ${M^{\prime }}_{1}=0$ and ${M^{\prime }}_{2}=\frac{2}{3}$. Although I have not been able to derive a closed-form solution for ${M^{\prime }}_{s}$, it is easily calculated numerically.

The aforementioned haplotype probabilities provide the key quantities for developing HMMs for advanced intercross populations. However, it should be noted that there are other approaches to handling such data. For example, Besnier *et al.* (2011) used a variance components model to analyze outbred chicken AIL data, with identity-by-descent probabilities calculated using a modified version of the method of Pong-Wong *et al.* (2001), for general pedigree data.

The aforementioned result for HS differs from that in Mott *et al.* (2000) and incorporated into the HAPPY software. They had assumed that the map expansion in HS was $\frac{7}{8}\left(s+2\right)$, whereas I show it to be $\frac{7}{8}\left(s-1\right)+3$. In the first three of generations with recombination, individuals are fully heterozygous, and so all recombination events can be seen; in the subsequent *s* − 1 generations, there is a 1/8 chance of homozygosity and so only 7/8 of recombination events can be seen.

Mott *et al.* (2000) further assumed that the transition probabilities along an HS chromosome are a function of genetic distance, but that requires knowledge of the map function. It is more direct to express the transition probabilities in terms of the recombination fraction at meiosis.

The green curve in Figure 1 displays the probability of a recombinant haplotype assumed in Mott *et al.* (2000) for HS with *s* = 10 when the map function corresponding to the gamma model with the level of crossover interference estimated for the mouse in Broman *et al.* (2002) is used. The probability is slightly smaller than that from my calculations; at *r* = 0.01, the equation in Mott *et al.* (2000) gives 0.099, whereas I obtain 0.103.

I have assumed an effectively infinite number of mating pairs at each generation. In practice, with a finite number of mating pairs, there will be some inbreeding and so an increased frequency of homozygosity and a decreased frequency of recombination. In addition, the individuals at the final generation will include siblings, and the relationships among individuals might be used to improve the genotype reconstruction. In practice, for computational efficiency, both the inbreeding and the relationships among individuals would probably be ignored in the genotype reconstruction, and with dense genotype data, there will be little loss of information.
